# Protein signaling and morphological development of the tail fluke in the embryonic beluga whale (*Delphinapterus leucas*)

**DOI:** 10.1002/dvdy.704

**Published:** 2024-03-17

**Authors:** L. M. Gavazzi, M. Nair, R. Suydam, S. Usip, J. G. M. Thewissen, L. N. Cooper

**Affiliations:** ^1^ School of Biomedical Sciences Kent State University Kent Ohio USA; ^2^ Musculoskeletal Research Focus Area, Department of Anatomy and Neurobiology Northeast Ohio Medical University Rootstown Ohio USA; ^3^ Wright State University Dayton Ohio USA; ^4^ Department of Wildlife Management North Slope Borough Utqiaġvik Alaska USA

**Keywords:** appendage, archaeocete, homology, mammalian evolution, novelty

## Abstract

**Background:**

During the land‐to‐sea transition of cetaceans (whales, dolphins, and porpoises), the hindlimbs were lost and replaced by an elaborate tail fluke that evolved 32 Ma. All modern cetaceans utilize flukes for lift‐based propulsion, and nothing is known of this organ's molecular origins during embryonic development. This study utilizes immunohistochemistry to identify the spatiotemporal location of protein signals known to drive appendage outgrowth in other vertebrates (e.g., Sonic Hedgehog [SHH], GREMLIN [GREM], wingless‐type family member 7a [WNT], and fibroblast growth factors [FGFs]) and to test the hypothesis that signals associated with outgrowth and patterning of the tail fluke are similar to a tetrapod limb. Specifically, this study utilizes an embryo of a beluga whale (*Delphinapterus leucas*) as a case study.

**Results:**

Results showed epidermal signals of WNT and FGFs, and mesenchymal/epidermal signals of SHH and GREM. These patterns are most consistent with vertebrate limb development. Overall, these data are most consistent with the hypothesis that outgrowth of tail flukes in cetaceans employs a signaling pattern that suggests genes essential for limb outgrowth and patterning shape this evolutionarily novel appendage.

**Conclusions:**

While these data add insights into the molecular signals potentially driving the evolution and development of tail flukes in cetaceans, further exploration of the molecular drivers of fluke development is required.

## Key Findings

- Cetacean flukes utilize similar proteins as found in the developing limb.
- This data provides insight into the evolution of a novel appendage.
- The exceptional fossil record for cetaceans allows us to make inferences on the developmental patterns associated with the origins of flukes in the Eocene.

## 1 INTRODUCTION

### 1.1 Elaborations of the soft tissues surrounding mammalian tails

Although the presence of a tail is common among mammals,^1^, ^2^, ^3^, ^4^ several lineages have independently evolved soft‐tissue elaborations that expand the functionality of the tail to facilitate life in the fluid habitats of the air and the water. As an example, bats are the only mammals capable of powered flight and most display a membrane of skin that connects the hindlimbs and encases the tail vertebrae. This thin and flexible elaboration of the soft tissues surrounding the tail plays a critical role in acting as a net for insect capture, and during flight it reduces drag on tail vertebrae, aids in flight control and take‐off, and controls pitching moment.^5^, ^6^, ^7^ The main propulsive organ in bats are the wings. In contrast, the obligatorily aquatic mammals [sirenians (manatees, dugongs) and cetaceans (whales, dolphins, porpoises)] evolved soft‐tissue elaborations of the tail that created a novel organ for lift‐based propulsion. The paddle‐shaped tail of manatees^8^, ^9^ and the triangular tail of dugongs likely share a common evolutionary origin, while the development of the tail flukes in cetaceans is an independent evolutionary event. The tail flukes of cetaceans act as a single propulsor and control surface.^10^, ^11^, ^12^ Although biologists have a thorough understanding of the anatomy of the flukes,^13^, ^14^, ^15^, ^16^, ^17^, ^18^, ^19^, ^20^ the biomechanical consequences,^11^, ^12^, ^18^, ^19^, ^21^ and the evolutionary history of tail flukes,^22^, ^23^, ^24^, ^25^ no data yet add insights into molecular mechanisms created the flukes.

### 1.2 Morphological elaboration of connective tissues in the tail flukes of cetaceans

**FIGURE 1:**
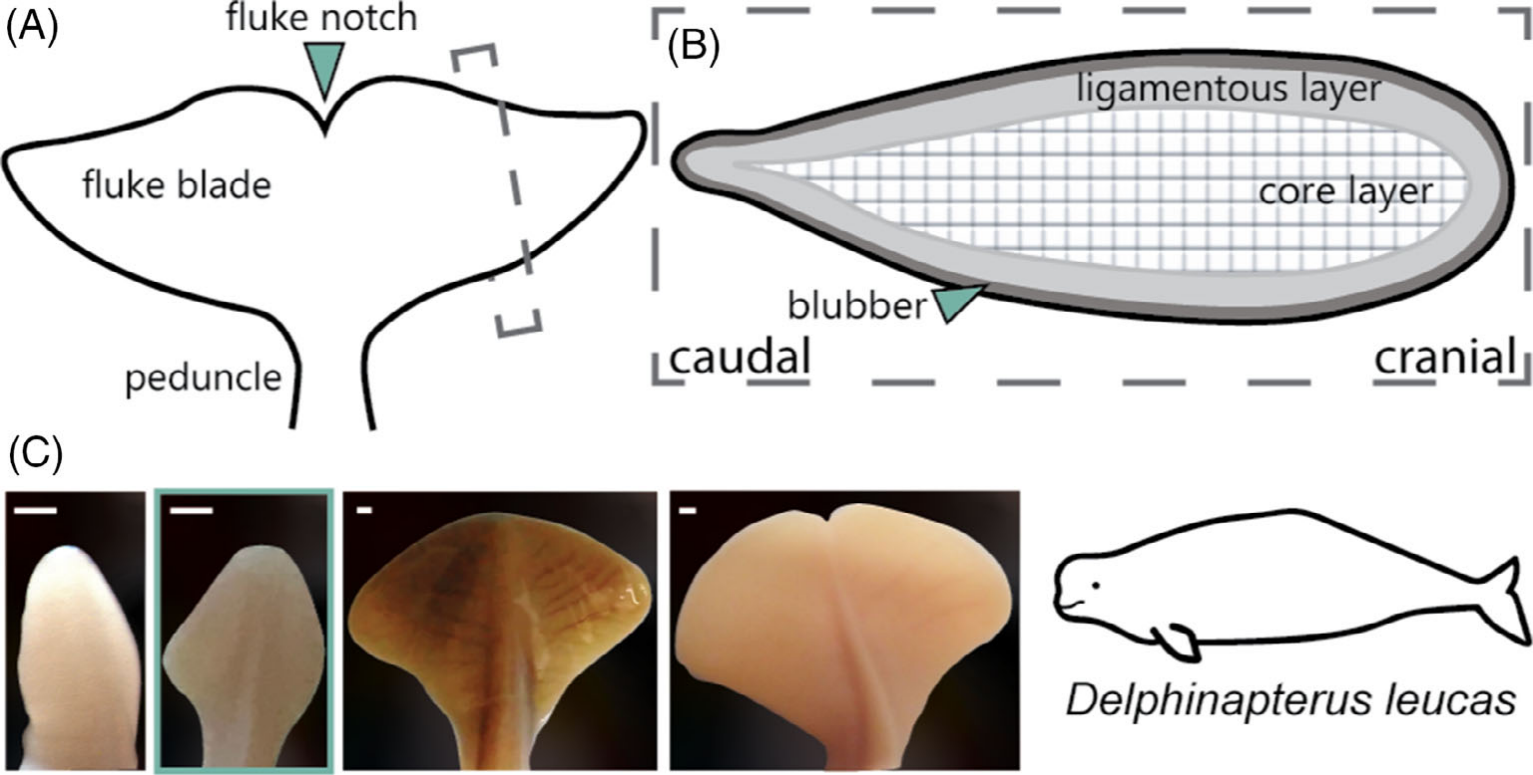
Identification of fluke anatomy and morphology using the beluga whale (*Delphinapterus leucas*) as the model taxon. (A) Basic anatomy of cetacean flukes in adults. (B) Internal morphology of the flukes of adult cetaceans in parasagittal section. (C) Photographs of embryonic fluke outgrowth through ontogeny based on embryonic specimens (from left to right): NSB‐DWM 2013LDL6F, 2011LDL11F and fetal specimens NSB‐DWM 2014LDL7F, 2012LDL10F. The blue box indicates the morphology of the sample selected for this study (NSB‐DWM 2009LDL9F). Scale bar = 1 mm.

The bilaterally symmetrical flukes of cetaceans are supported by caudal tail vertebrae and dense connective tissues that are enveloped by skin (Figure 1A). The two triangles of the adult fluke are located lateral to the caudal vertebrae with a notch in the midline (Figure 1A). Underneath the smooth epithelial layer covering the fluke, the connective tissue core has two layers: (1) the outer ligamentous layer of collagens and (2) the internal core layer made of a stiff meshwork of collagens (Figure 1B).^15^, ^18^, ^20^ Also contained within the fluke is an elaborate vascular network. Epaxial and hypaxial tendons encircle the fluke vertebrae and there is no musculature within the fluke blades.

### 1.3 Morphological development of the tail fluke

Although the vertebrae of the cetacean tail develop similarly to other mammals, the soft‐tissue flukes undergo several transitions in shape during prenatal development. Initially, mesenchymal tissues differentiate and proliferate into collagens, and outgrowth creates a lanceolate or diamond‐shaped structure.^14^, ^26^, ^27^ As outgrowth continues, the flukes transiently take on spade‐like and heart‐shaped morphologies until finally forming two bilaterally symmetrical triangle‐shaped structures (Figure 1C).^14^, ^27^ Molecular events directing this process are unknown, but these patterns of outgrowth were hypothesized to be consistent with and related to limb development.^26^, ^28^

### 1.4 Appendage patterning and outgrowth

The field of evolutionary developmental biology reveals that outgrowth and patterning of limbs, fins, and genitals are under overlapping genetic pathways.^29^, ^30^, ^31^, ^32^, ^33^, ^34^ Outgrowth of these appendages requires cross‐talk in gene expression between the embryonic epithelium and underlying mesenchyme. Previous studies of gene expression have found functional connections between limb and fin cascades^33^, ^34^, ^35^, ^36^, ^37^, ^38^ and similar gene expression patterns between the mammalian limb and genital tubercle.^31^, ^39^, ^40^, ^41^, ^42^ Based on this fundamental literature, this study utilizes the embryonic tail of a beluga to understand whether outgrowth and patterning of the fluke blades resembles the molecular cascades driving limb, fin, and/or genital development.

### 1.5 Contrasting developmental cascades associated with appendage development

Outgrowth of limbs and fins are similar in their spatiotemporal gene expression patterns and associated functions. Along the distal ends of developing limbs and fins, a thickened region of the ectoderm (apical ectodermal ridge [AER]) secretes fibroblast growth factors (FGFs 4, 8, and 10) that largely cause the underlying tissues to proliferate and thereby direct outgrowth of these appendages.^40^, ^43^, ^44^, ^45^, ^46^, ^47^, ^48^, ^49^ Early in development, teleost fins and tetrapod limbs are nearly identical in FGF signaling.^32^, ^44^, ^50^, ^51^, ^52^, ^53^

FGFs also coordinate with a signaling center within the mesenchyme, the zone of polarizing activity (ZPA), that patterns the appendage along an anterior–posterior axis (e.g., determining digit number) through the expression of Sonic Hedgehog (SHH).^54^, ^55^, ^56^, ^57^, ^58^ Where the AER and ZPA function as the two major signaling centers of the fin and limb, the major organizing center of the genital tubercle is the distal urethral epithelium (DUE).^31^, ^41^, ^42^, ^59^, ^60^ This epithelium primarily secretes SHH and there are high levels of the morphogen found both within the DUE and along the midline of the genital tubercle. In the genitals, it is hypothesized the SHH patterns the appendage and promotes outgrowth, similar to the roles of both the AER and ZPA in other appendages.^29^, ^39^, ^40^ Data gathered from in‐situ hybridization experiments show that *FGF8* is expressed in a very small midline region within the DUE^31^, ^42^ and the mRNA (but not proteins) of *FGFs* are present in a small area of the midline of developing genitals.^29^, ^31^, ^59^ This study tested for the presence and location of FGF and SHH proteins within the developing flukes of the beluga whale as a first step in establishing if a limb, fin, or genital paradigm is employed.

In addition, this study tested the spatial signaling patterns of other key proteins associated with limb development. The dorsoventral axis of the limb is specified by wingless‐type family member 7a (WNT7A) and LIM homeobox transcription factor 1 (LMX1) on the dorsal aspect while the ventral side is specified by the transcription factor Engrailed‐1 (EN1).^61^, ^62^, ^63^, ^64^ There is robust evidence suggesting that the midline of these conflicting dorsoventral signals indicates the developing limb where the AER should begin to form.^65^, ^66^, ^67^, ^68^, ^69^ There is also evidence that teleost fins express WNT7A despite being dorsoventrally symmetrical appendages.^70^ We chose to investigate WNT7A in the developing beluga flukes given the ubiquity of this protein in both fins and limbs.

Bone morphogenetic proteins (BMPs) are implicated throughout limb patterning as both upstream and downstream targets of the FGFs and SHH. In particular, BMPs are considered critical for the formation of the digits via apoptosis of the interdigital tissues of the tetrapod limbs.^71^, ^72^ In animals that maintain webbed appendages, such as ducks, bats, and cetaceans, this BMP expression is present in conjunction with GREMLIN (GREM) and FGF activity in the interdigital zone^73^, ^74^ and the effects of BMP activity associated with apoptosis are reduced in these taxa, preserving the soft‐tissue. Like webbed appendages, the fins do not experience a major apoptotic event and there is evidence of a GREM–FGF–SHH feedback loop found in the formation and growth of the midline fins, suggesting that BMP is still critical to median fin development.^44^, ^53^

As an appendage, the cetacean tail flukes originated long after Lower Cambrian fin evolution,^75^, ^76^, ^77^ Devonian tetrapodal limb transition,^78^, ^79^, ^80^ and emergence of the amniote penis.^81^, ^82^ With the first evidence for flukes in the Eocene approximately 38 MYA,^22^, ^83^ the morphological and evolutionary novelty of the cetacean flukes compared to fins and limbs allows key insight into the pathways that drive appendage formation. Cetaceans have long been regarded as an iconic organism for extreme mammalian evolution, and the last few decades of molecular research have provided a crucial understanding of the unique developmental processes that govern extremity development in both generalized and highly derived mammals. Here, we use immunohistochemistry to investigate the spatial association of appendage patterning proteins within the tail and fluke tissue of an embryonic beluga whale. The data presented here provide some insight into the initial development of the flukes, informing our understanding of the fundamental building blocks necessary for the evolution of a novel appendage.

## 2 RESULTS

### 2.1 Fluke embryology and cellular morphology

Three prenatal beluga flukes were stained with hematoxylin and eosin (H&E) and a modified Mallory's trichrome^84^ to assess internal cellular morphology. In this Mallory's trichrome the erythrocytes stain red, undifferentiated connective tissue is blue, and differentiated connective tissue is blue‐purple. Metabolically active cells will take on an orange hue throughout the entire cell, allowing for the identification of active growth and differentiation zones. The three beluga embryos were assigned a corresponding Carnegie Stage based on the criteria set in Gavazzi et al.^27^ That study used external morphological characteristics to determine a relative developmental trajectory for cetaceans, and the lower numbers correspond to earlier periods of prenatal development. All three beluga samples [NSB‐DWM 2009LDL9F (CS 19), 2014LDL7F (CS 20), and 2012LDL10F (fetal)] had three different fluke morphologies from diamond‐shaped to triangle‐shaped, respectively.

**FIGURE 2:**
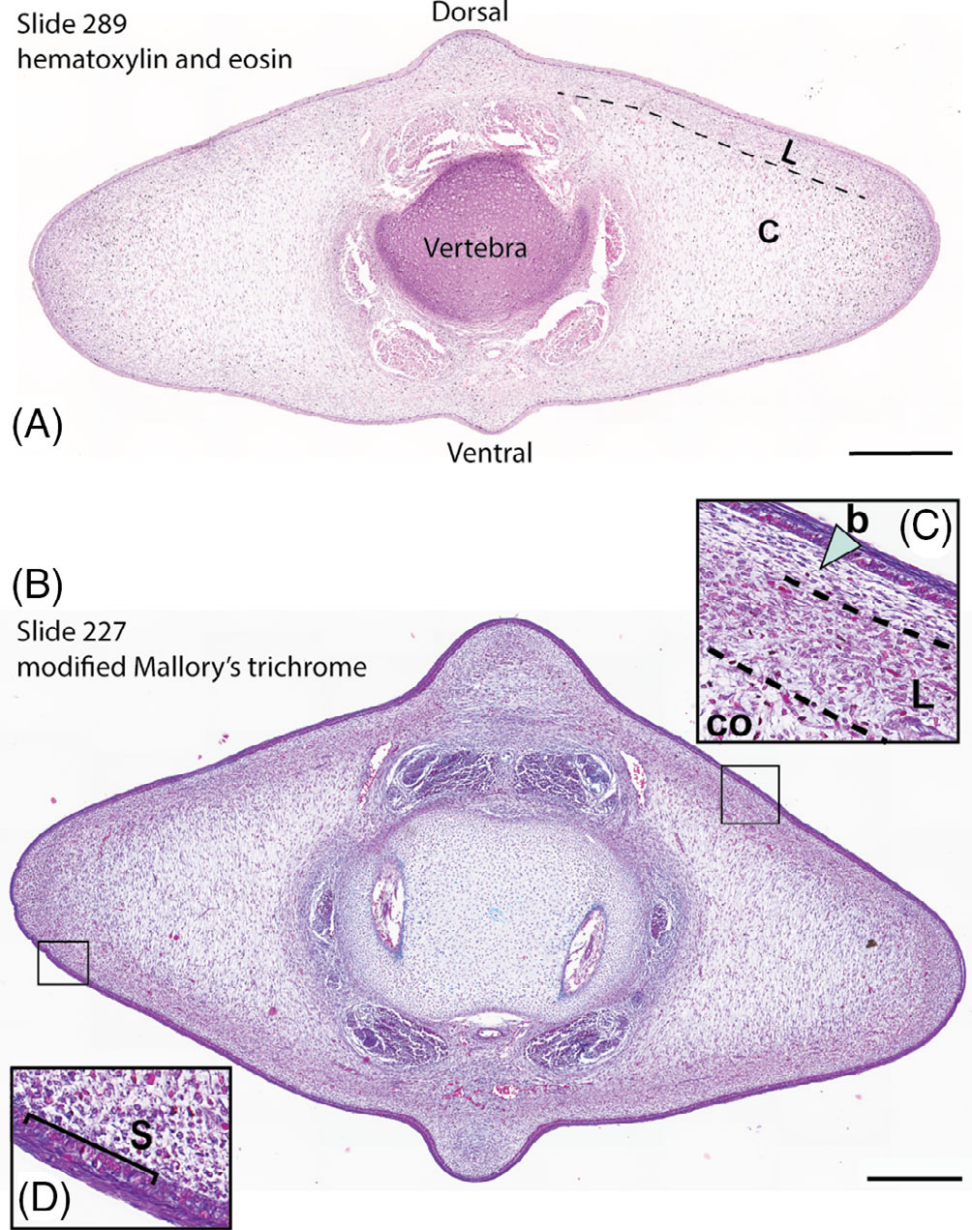
Cross‐sections of fluke tissue from NSB‐DWM 2009LDL9F. (A) Hematoxylin and eosin‐stained section. (B) Section stained with a modified Mallory's trichrome. (C) b—blubber, L—ligamentous layer, Co—core layer. (D) S—Stratum basale. Scale bar = 0.5 mm.

Initial staining of the embryonic fluke tissue with H&E reveals cellular morphology that is generally similar to that described in adult cetaceans.^13^, ^14^ The cross‐section in Figure 2A shows that the prenatal flukes are predominately comprised of mesenchymal cells covered by a simple squamous epithelium. This mesenchyme and the corresponding extracellular matrix have two different orientations: the outer layer appears to span the tissue craniocaudally and the inner layer has a dorsoventral orientation. This differentiation roughly aligns with the ligamentous and core layers of the post‐natal cetacean flukes.^15^, ^19^ Within the flukes, there is a greater density of nuclei at the lateralmost edges, suggesting that this may be a highly proliferative area. At this point in development in the smallest beluga embryo, the flukes are diamond‐shaped and will transition to a spade shape thereafter.^27^ The skin of the flukes is thin. The vertebra is still a cartilaginous anlage surrounded by epaxial and hypaxial tendon precursors.

Within the flukes of the smallest beluga, NSB‐DWM 2009LDL9F, the presumptive ligamentous and core layers are highly contrasted with Mallory's stain. What we described as the ligamentous layer within the H&E‐stained section of the flukes appears to be subdivided into two different cell types (Figure 2C). Just deep to the skin, the lamina propria is comprised of purple cells, indicating a differentiated connective tissue, with little extracellular matrix. The deeper mesenchymal layer is orange‐purple, which suggests highly active and proliferative cells. It is possible that these layers are the precursors to the blubber (b) and ligamentous layer (L), respectively. Both of these cell layers are superficial to the core layer (Co). The core layer has purple staining mesenchymal tissue with long extracellular projections running dorsoventrally. Furthermore, there is a greater density of connective tissue cells in the ligamentous layer than in the core layer overall, contributing to the darker staining pattern. At the lateral edge of the fluke, there are a high number of orange‐purple cells, indicating that this region is one of increased cellular activity (Figure 2D). The skin of the flukes is purple and all nuclei of the stratum basale (S) are orange‐purple, which is indicative of differentiated tissue and a stratum basale that is synthesizing elevated levels of mRNA and protein. Throughout the connective tissue, erythrocytes can be seen via their bright red staining.

**FIGURE 3:**
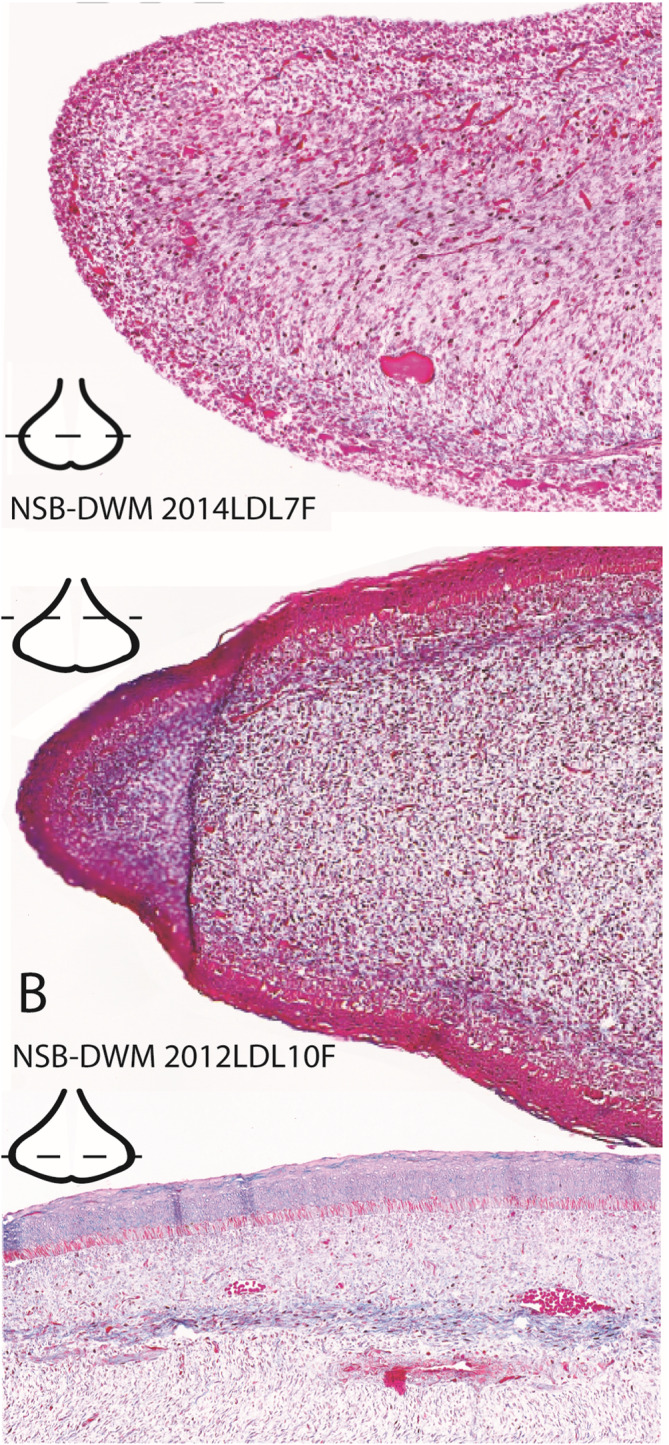
Modified Mallory's trichrome staining of CS‐20 NSB‐DWM 2014LDL7F (A) and fetal specimen NSB‐DWM 2012LDL10F (B and C). (A) and (B) are enlarged images from the lateral edge of the left fluke blades. (C) is an enlarged image of the dorsolateral region of the left fluke blade. Level of section is indicated on the fluke illustrations.

The NSB‐DWM 2014LDL7F flukes (Figure 3A) are highly vascularized with blood vessels visible throughout the tissue. In the central portion of the fluke, differentiation of the core layer has been initiated. There is additionally a dark blue/purple band of tissue just deep to the first layer of connective tissue, which also suggests differentiation of the mesenchyme. The skin of this specimen had peeled away prior to fixation, so the degree of epithelial proliferation or staining is unknown. As previously found in NSB‐DWM 2009LDL9F, there is a highly dense region of nuclei at the lateral edge of the fluke blade, suggesting that this region is still proliferating.

NSB‐DWM 2012LDL10F (Figure 3B,C) shows a similar pattern to Figure 3A with differentiation at the innermost core layer in the fluke blades. The tissue just deep to the epithelium has a blue hue, suggesting further differentiation of these tissues. The skin in a more cranial section of the flukes, towards the peduncle, is red (Figure 3B) but shows evidence of differentiation more caudally (Figure 3C). Though this specimen is similar in morphology to the perinatal fluke shape, the lateral edges of this tissue are still densely packed with nuclei and appear to maintain a state of high proliferation when compared to the rest of the connective tissue in these sections, as evidenced by the red staining of the cells in the lateral region.

### 2.2 Protein signaling in the beluga flukes

Of the three flukes sectioned and stained with trichrome, we selected the best‐preserved specimen, NSB‐DWM 2009LDL9F for further investigation of protein signaling in the flukes via immunohistochemistry. We chose to investigate the following proteins based on their known roles in appendage patterning and outgrowth: FGF8, FGF10, SHH, WNT7A, GREMLIN, and BMP4. BMP4 was not detected in any of the samples. We report the results for the five other proteins tested below.

**FIGURE 4:**
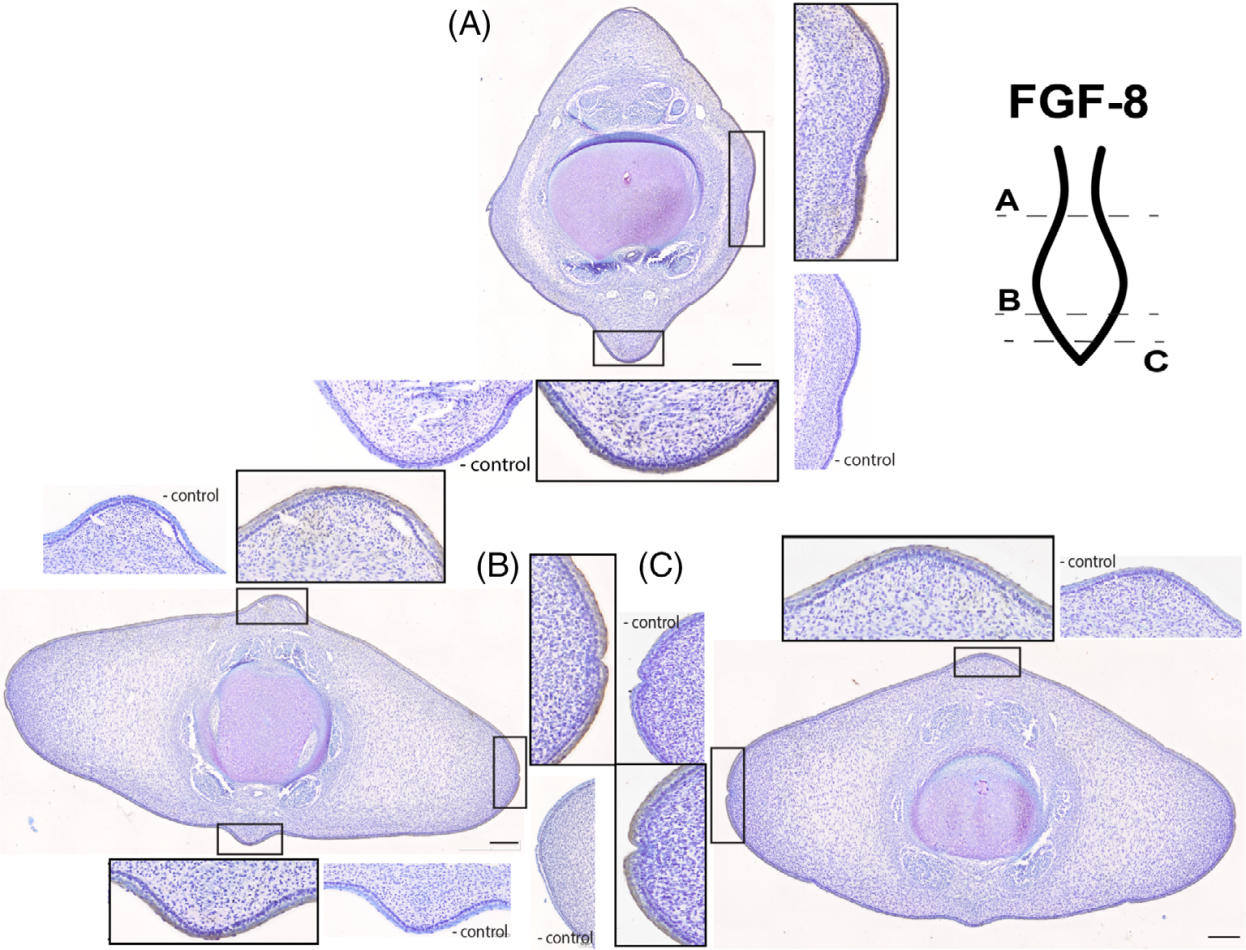
Fluke tissue from NSB‐DWM 2009LDL9F stained with FGF8 antibody and counterstained with 0.01% thionin. Level of section (A–C) and relationship to overall soft‐tissue flukes indicated on illustration. Scale bar = 250 μm.

**FGF8** (Figure 4): In the area just caudal to the peduncle there is consistent staining for the FGF8 antibody (Figure 4A). The protein is found circumferentially in the outermost epithelium, with no stain found deep to the basement membrane. In the distal third of the fluke, caudal to the widest point, the chromogen staining for FGF8 is comparatively darker (Figure 4B). The staining of the epithelium encapsulates the entirety of the epidermis. The caudalmost section of the fluke has light staining of the outermost layer of the epithelium (Figure 4C). There is no asymmetry to the staining pattern and there is no stain within the mesenchyme or other connective tissues.

**FIGURE 5:**
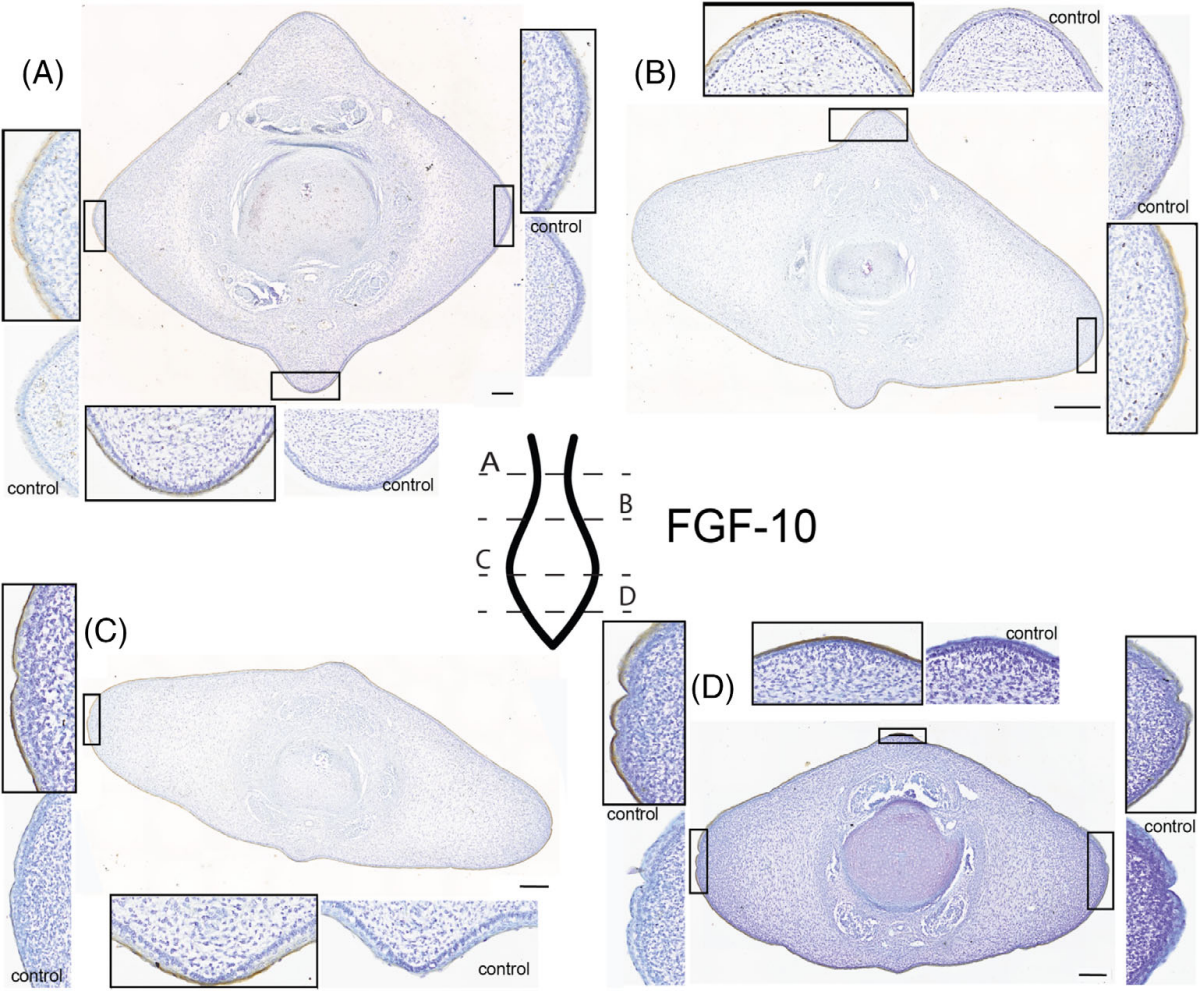
Fluke tissue from NSB‐DWM 2009LDL9F stained for FGF10 antibody and counterstained with 0.01% thionin. Level of section (A– D) indicated on fluke illustration. Scale bar = 250 μm.

**FGF10** (Figure 5): The staining pattern for FGF10 on these tissue sections closely matches the staining pattern seen for FGF8. In the first section just caudal to the peduncle (Figure 5A) the outermost epithelium demonstrates binding for the FGF10 antibody except for the right lateral fluke, which may be an artifact. Along the proximal half of the flukes (Figure 5B), there is dark staining for the FGF10 antibody within the outermost squamous layer of epithelium. This is consistent circumferentially. At the lateral fluke edges, the staining for FGF10 comprises the entire squamous layer of the epithelium, though the skin is also much thinner in this region. There is no FGF10 chromogen found in the stratum basale of the epithelium. At the widest part of the fluke, the staining found in FGF8 and in the previous FGF10 section matches (Figure 5C). Intense chromogen staining is found in the outermost epithelium. There is no expression deep to the basement membrane in any portion of the connective tissues. At the tip of the tail (Figure 5D), FGF10 is highly expressed in the epithelium in a pattern identical to Figure 5B,C.

**FIGURE 6:**
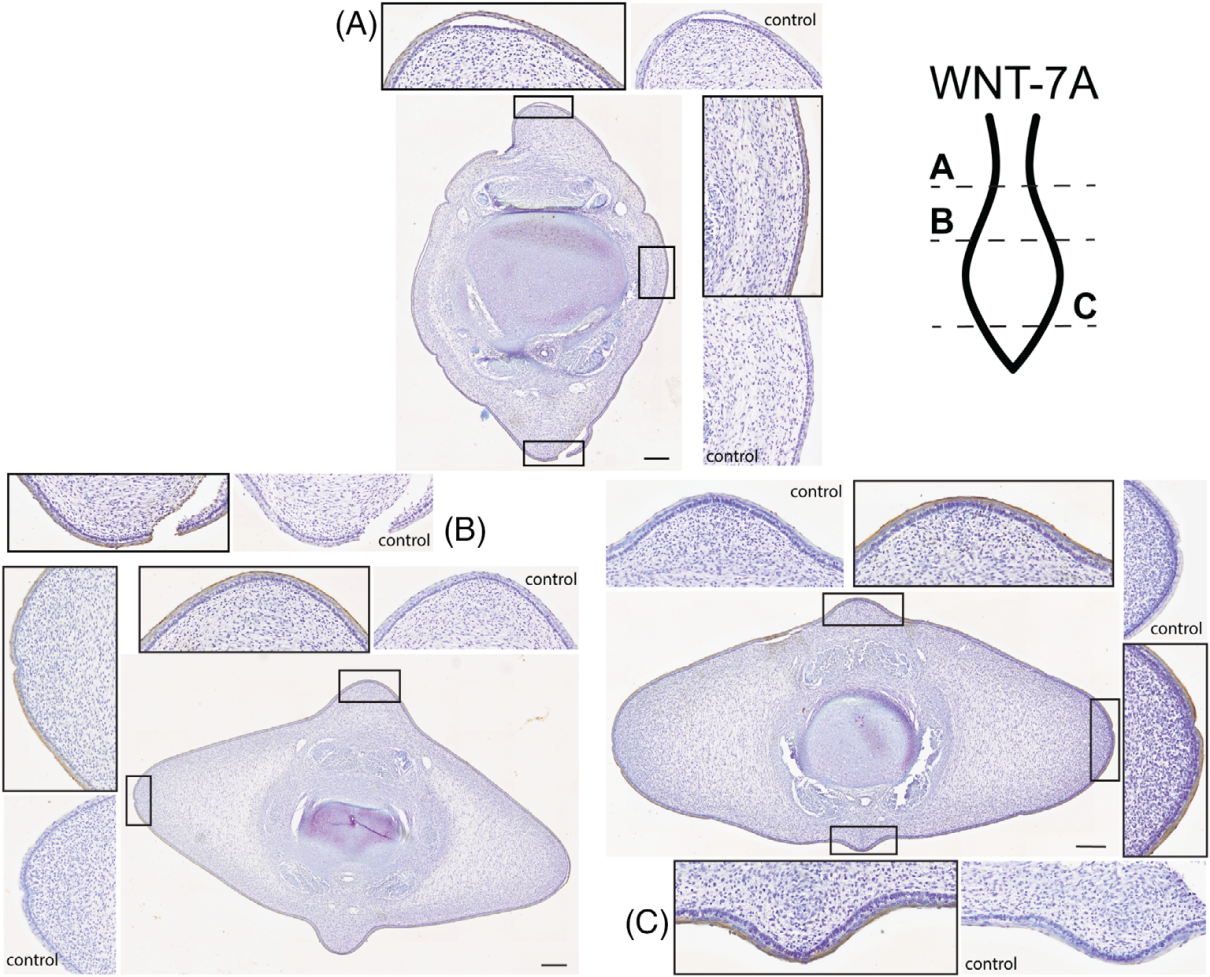
Fluke tissue from 2009LDL9F stained for WNT7A antibody and counterstained with 0.01% thionin. Level of section (A–C) indicated on fluke illustration. Scale bar = 250 μm.

**WNT7A** (Figure 6): At the level of the peduncle (Figure 6A), staining for the WNT7A antibody can be found in the dorsal, ventral, and lateral epithelium. There does not appear to be any variation in staining pattern between these regions, and the staining appears more concentrated within the squamous and superficial portion of the epithelium. In the cranial half of the diamond‐shaped flukes, staining is found circumferentially in the epithelium (Figure 6B). There appears to be a stronger antibody staining in the ventral keel of the fluke section. This is opposite of what is expected for the standard limb WNT7A signaling pattern, where this protein is found in the dorsal epithelium. In the distal tip of the fluke (Figure 6C) the same pattern is shown but does not demonstrate the dorsoventral asymmetry found in Figure 6B. Here, the epithelium is darkly stained for the WNT7A antibody in the dorsal and ventral keels as well as the lateral flukes.

**FIGURE 7:**
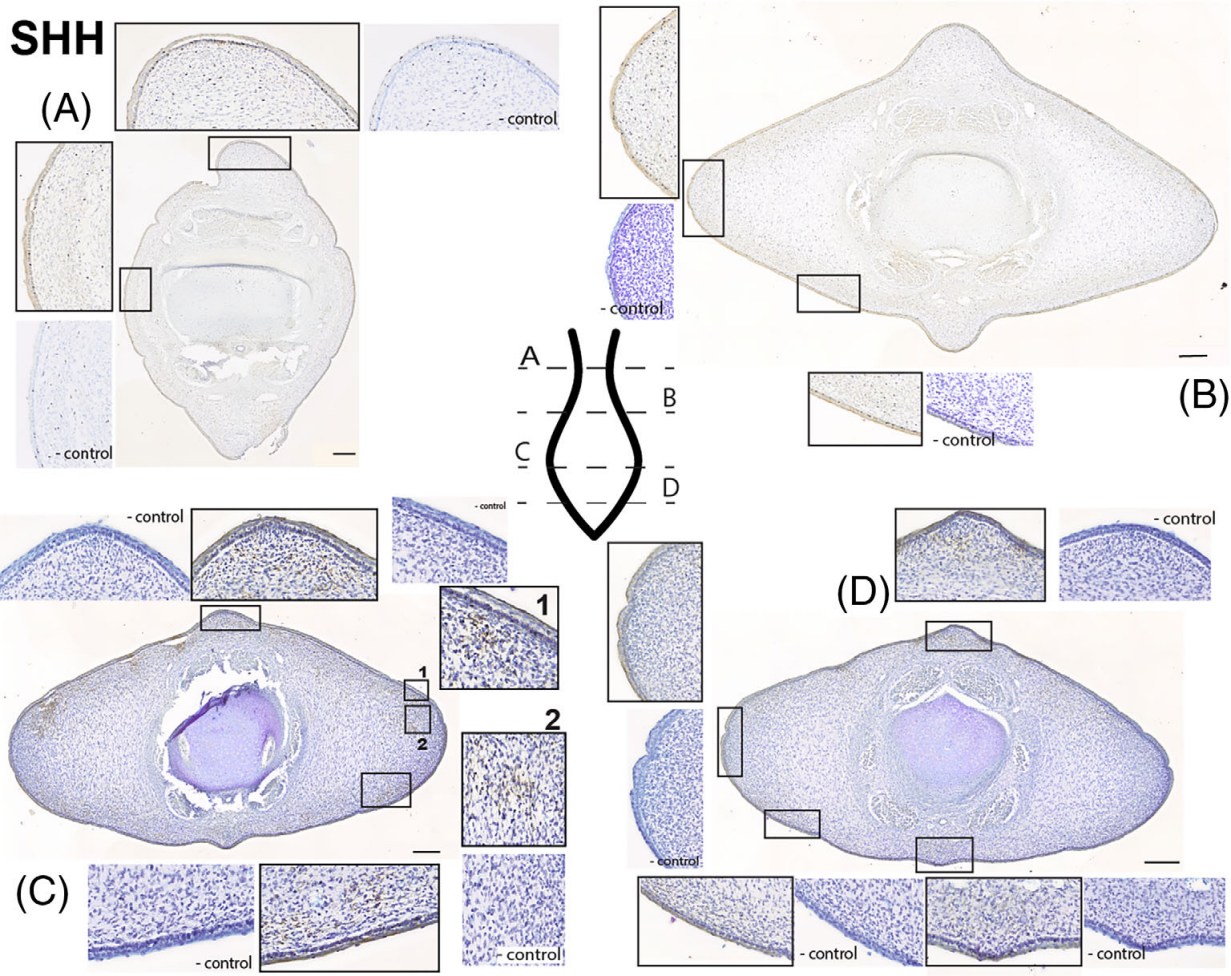
Fluke tissue from NSB‐DWM 2009LDL9F stained for SHH antibody and counterstained with 0.01% thionin. On section C, 1 and 2 are potential SHH hotspots. Level of section (A– D) indicated on fluke illustration. Scale bar = 250 μm.

**SHH** (Figure 7): At the peduncle, SHH signaling is found in the epithelium and in the ligamentous layer of mesenchyme (Figure 7A). The pattern of staining in these two regions is distinct: within the skin the stain is dark and consistent and in the ligamentous layer the SHH antibody binds in a “speckled” or scattered pattern. Punctate brown staining is clear throughout the superficial mesenchyme and does not appear to show a mediolateral or dorsoventral asymmetry. Staining in the first 1/3 of the fluke (Figure 7B) matches the patterns found in the peduncle.

Close to the tip of the tail (Figure 7C), there is staining for the SHH antibody in both the epithelium and within the mesenchymal tissue just deep to the epithelium. There is consistent antibody staining within the skin, presence of SHH signaling in the squamous cell layer, and speckled staining within the presumptive ligamentous layer of mesenchymal tissue. As found in other sections, this pattern is circumferential and does not show any major dorsoventral or mediolateral variation. On the right dorsolateral surface are two patches of staining that do not correspond to the common pattern found elsewhere in the tissue; one unusual signaling region is found just deep to the epithelium (1) and the other is deeper still within the densely nucleated mesenchyme of the right lateral fluke (2) within the core layer of the fluke. These ‘hotspots’ appear to be bilateral; however, the left lateral fluke also has tearing of the epithelium in this region, making precise interpretation of the stain pattern difficult.

In the caudalmost section of the fluke (Figure 7D), epithelial SHH staining is consistent with the other sections. While there is the presence of SHH signaling and chromogen staining in the mesenchyme, it appears limited to the ligamentous layer, including the dorsal and ventral keels. This pattern matches the staining described in Figure 7C; however, the intensity of the stain is greatly reduced compared to that earlier section. Notably, the ‘hotspots’ found in the previous section are not present in this section.

**FIGURE 8:**
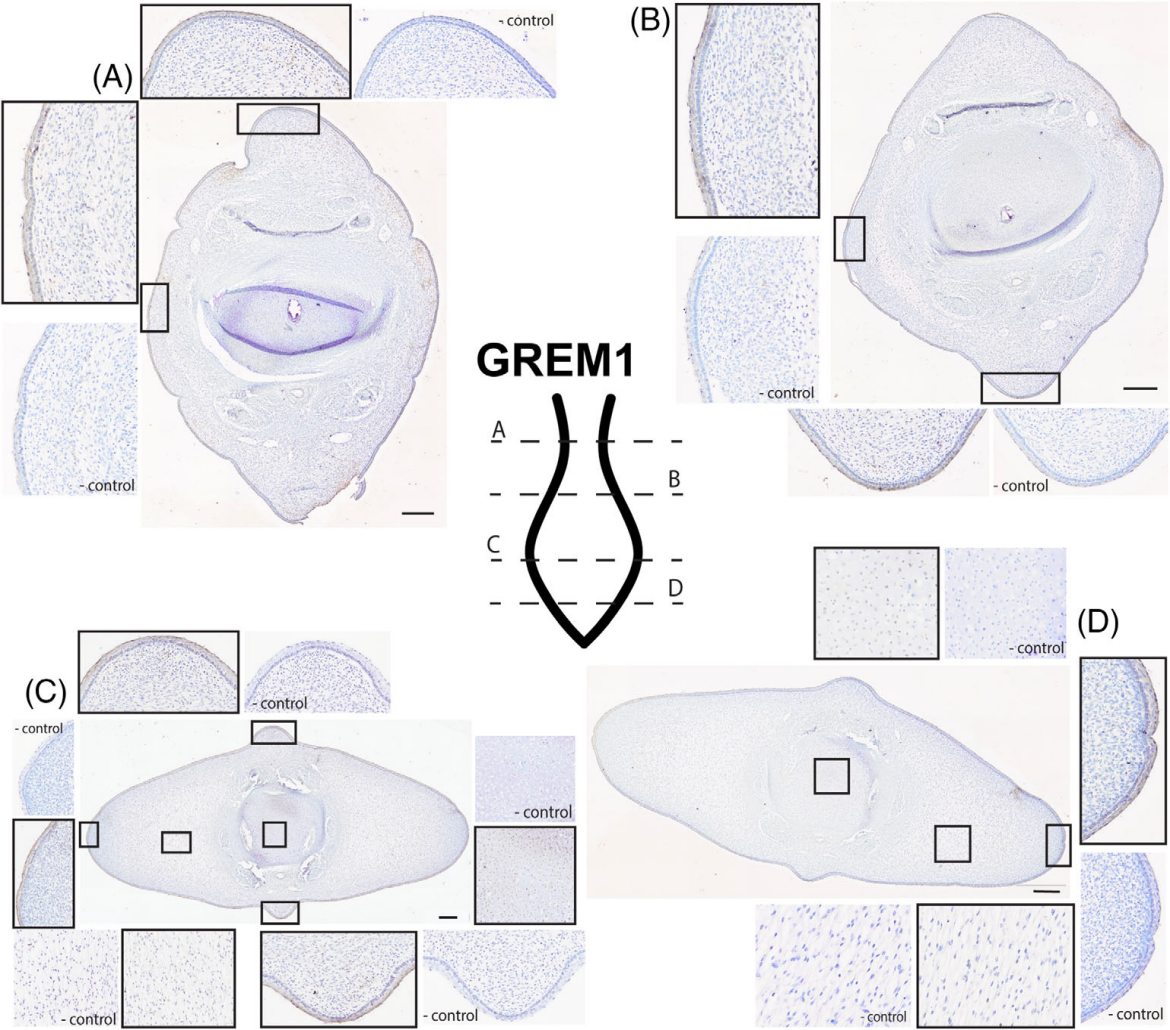
Fluke tissue from NSB‐DWM 2009LDL9F stained for GREM antibody and counterstained with 0.01% thionin. Level of section (A– D) indicated on fluke illustration. Scale bar = 250 μm.

**GREMLIN** (Figure 8): Just cranial to the peduncle, there is staining for GREM in the squamous portion of the epithelium (Figure 8A). Like SHH, there is also some punctate staining within the superficial layer of connective tissue just deep to the skin. The speckling of this GREM staining is more diffuse than that seen in SHH. The cranial half of the flukes (Figure 8B) appears to be nearly identical to that of Figure 8A. The only considerable difference between these two sections is the amount of staining within the connective tissue. There is very little staining present in the mesenchyme of Figure 7B, and many of the patches that are present appear in proximity to tears within the tissue. At the widest part of the flukes, staining for the GREM antibody is found in the outermost layer of the epithelium, in a punctate pattern deep to the stratum basale, and in the cartilage of the vertebra (Figure 8C). This protein is the only one found in the deeper layers of the flukes and within the vertebral body. There appears to be some faint staining for GREM in the core layer of the flukes as well, as the tissue exposed to the antibody has a darker hue than the control tissue. Towards the caudal tip of the developing flukes (Figure 8D), the staining is similar to the previous section, Figure 8C. There is antibody staining in the epithelium and cartilage, with faint gray/brown pigmentation in the core layer. There is little to no GREM staining within the outer layer of connective tissue.

Our results localized FGF8, FGF10, and WNT7A exclusively to the epidermis of the flukes. Both SHH and GREMLIN showed expression within the epithelium and mesenchymal connective tissue of the flukes. SHH localized to the ligamentous layer of the fluke connective tissue while GREMLIN was found within the deeper core layer of tissue and within the cartilage. In summary, all five proteins investigated in this study showed staining in the epithelium, particularly in the squamous layer of the skin, and only two proteins showed staining within the mesenchymal fluke tissue.

## 3 DISCUSSION

### 3.1 Cetacean limb development

Protein signaling during embryogenesis in cetaceans is best understood in the pan‐tropical spotted dolphin (*Stenella attenuata*). The ontogeny of this taxon has been documented and described from early somitogenesis to the fetal period.^85^, ^86^ Gene expression hypotheses based on visual examination of *Stenella* embryos^87^ have been further tested via immunohistochemistry on both the forelimb and hindlimb buds.^73^, ^88^ These studies demonstrate that cetacean limbs initially form using the common limb developmental signaling cascade but diverge early in development to form cetacean‐specific traits. Key proteins related to limb development have been identified in *Stenella* at a similar developmental age to the beluga embryo utilized for this study.^27^, ^73^, ^88^

In the forelimb, *Stenella* maintains FGF8 signaling in the AER for a prolonged period of time when compared to mice and pigs, an artiodactyl relative of whales. Furthermore, where other mammals express BMPs within the interdigital tissues to sculpt the digits within the acropodium, the interdigital zone of the cetacean forelimb co‐expresses FGF8 and GREM to help maintain this webbing for the soft‐tissue flipper.^73^ Concurrently, the hindlimbs of cetaceans initiate like a typical mammalian limb bud except for a lack of SHH protein signaling from the ZPA. This aberration, which would lead to the loss of anteroposterior axis specification and the requisite feedback loops necessary to maintain limb proliferation, is implicated in the eventual cessation of limb cell proliferation in *Stenella*.^88^

Speculation has suggested that the outgrowth of tail flukes has an intimate association with the regression of the hindlimb buds,^28^ and may develop using an appendage‐like signaling regime,^26^, ^28^ which here we test empirically. We used immunohistochemistry to identify and localize some of the common protein signals associated with morphogenesis and outgrowth of appendages during embryonic development. Of the three appendages (fins, limbs, and genitals), we hypothesized that cetacean fluke development most closely resembles the protein‐staining patterns found in limb development. Fin development is restricted to non‐tetrapodal taxa, and the formation of the limb from the fin suggests that the initial conditions needed to form a functioning fin are likely modified within tetrapods. The genital tubercle is different enough from the limb and fin that it has independently evolved distinct expression patterns after the initial exaptation of the limb/fin signaling cascade. It seems unlikely that the flukes of this beluga would then take signaling cues from this highly derived appendage.

### 3.2 Protein signaling in the soft‐tissue flukes

Initial staining of the flukes of a beluga embryo using H&E revealed that the flukes are largely comprised of mesenchymal tissue that is rudimentarily divided into the ligamentous and the core layers. We also see a secondary layer within the presumed ligamentous layer that may be the developing blubber (Figure 2C). Additionally, there is some evidence for vascularization of the flukes, as small blood vessels can be seen throughout the trichrome‐stained section, indicated by bright red blood cells. This particular trichrome protocol is designed to identify highly active cells,^84^ which were found in the lateralmost portions of the flukes and also within the stratum basale of the epithelium. The prevalence of highly active cells at the epithelial/mesenchymal boundary correlates with our protein findings; all five of the antibodies tested had some level of binding within the epithelium.

**FIGURE 9:**
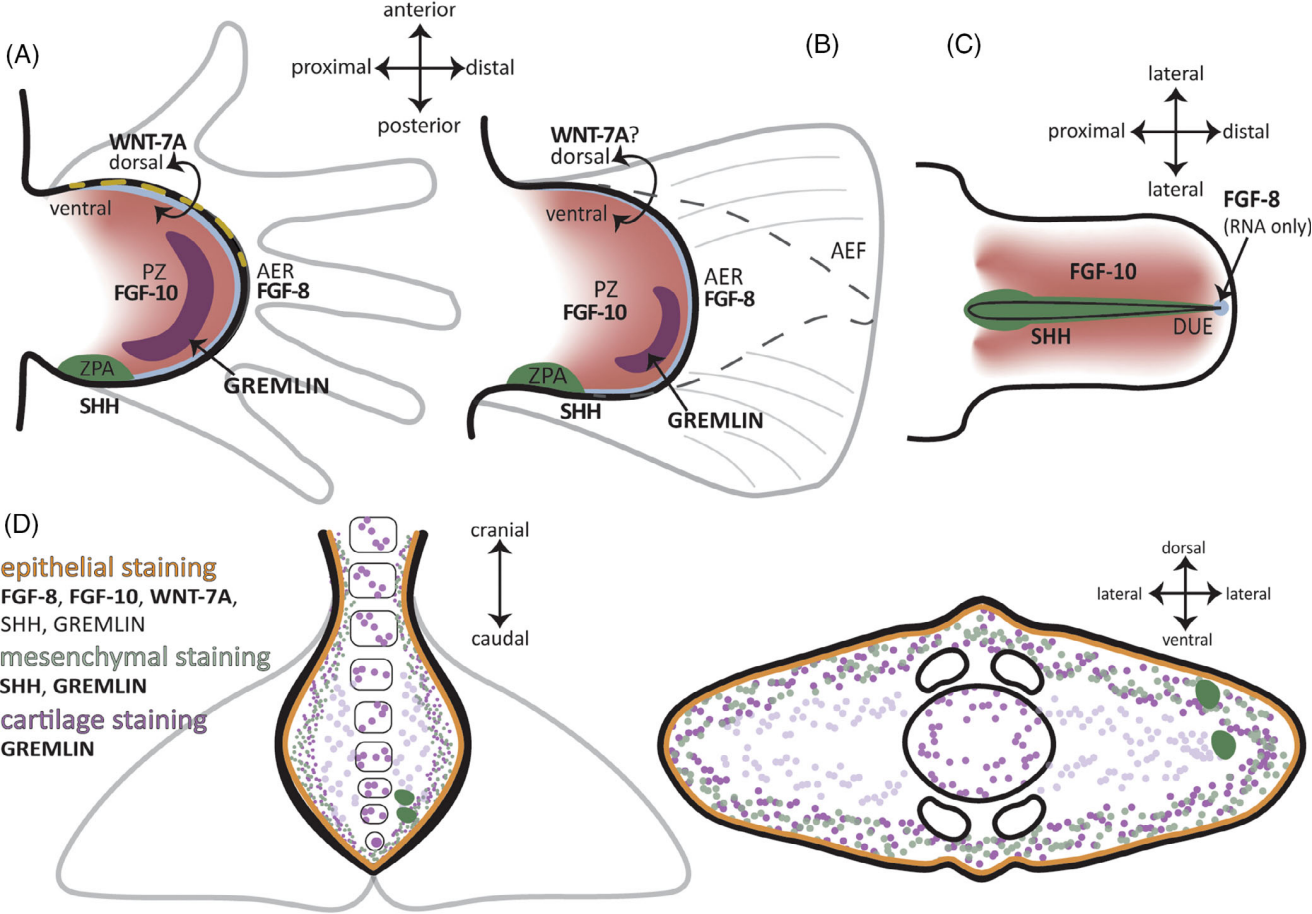
Illustrations of protein signaling during limb (A) (embryonic day 11.5), fin (B) (~20 h post fertilization), and genital tubercle (C) (embryonic day 13.5) development for comparison with embryonic beluga fluke immunohistochemical (IHC) results (D). Illustrations not to scale. AER, apical ectodermal ridge; DUE, distal urethral epithelium; ZPA, zone of polarizing activity.

We used immunohistochemistry to determine the presence and location of common proteins associated with appendage patterning and outgrowth. These protein data, when analyzed together, indicate that some of the proteins critical for appendage development have been exapted for the formation of the flukes. First, we assessed the proteins involved in fin and limb proximodistal outgrowth, FGF8, and FGF10. In the flukes, FGF8 is exclusively present within the epithelial tissue. This pattern matches the data found for FGF8 within the AER or fold in other taxa, supporting the appendage hypothesis (Figures 4 and 9). In the genital tubercle, *FGF8* mRNA has been reported in a small region of the DUE, though these data could not be replicated at the protein level.^31^, ^40^, ^59^ For the protein FGF10, the staining pattern closely matches FGF8. Typically, the developing limb of tetrapods shows FGF10 expression in the mesenchyme underlying the AER,^47^, ^89^, ^90^, ^91^ not concurrently in the same tissue as seen in this beluga (Figure 9). While there is this distinct heterotopy between a generalized limb paradigm and the flukes, the presence of FGF10 in the developing epithelium at the same location and tissue level of FGF8 suggests that FGF10 is likely serving a similar role as found in the developing appendage.

Prenatal fluke skin is divided into the stratum basale and an overlying stratified squamous epithelium. There is no evidence of epithelial thickening or stratified cuboidal/columnar cell layer in the skin, both of which can be morphological markers of an AER. Previous evidence has shown that AER morphology varies greatly across tetrapods, yet it is the signaling patterns involving FGF8 that drive the function of the ectoderm.^92^ Although beluga fluke tissue does not demonstrate any morphological indicators of an AER, presence of FGF8 in the epithelium suggests that FGF8 plays a similar role in the flukes as in the appendages.

Like both of the FGF proteins, the WNT7A protein was found circumferentially in the epithelium. While the tissue layer here is consistent with the appendage hypothesis, the lack of asymmetrical signaling is not. This pattern seen in the flukes does not lend itself to dorsoventral patterning as seen in the tetrapod limb, where WNT7A is only found in the dorsal ectoderm^62^, ^63^, ^64^, ^66^, ^93^, ^94^, ^95^ (Figures 6 and 9).

Given that the flukes are a dorsoventrally symmetrical organ, this staining pattern may relate to a lack of dorsoventral patterning. If the cetacean flukes have exapted the vertebrate limb signaling pattern, then the WNT7A expression pattern was likely modified to maintain dorsoventral symmetry within the flukes as opposed to the limb. Experimental knockout of *LMX1B* mRNA, a gene expressed in the ventral mesenchyme that maintains dorsoventral signaling boundary, expands the ectodermal expression of *WNT7A* to encompass both the dorsal and ventral ectoderm of the modified limb bud.^63^, ^64^, ^96^ Additionally, reduction of either *LMX1B* or *EN‐1* mRNA expression in animal models leads to the development of a limb that is more dorsoventrally symmetrical than wild‐type counterparts.^63^, ^64^, ^93^, ^97^ This experimental data using mouse models suggest that a similar modification to the LMX1B/EN‐1/WNT7A signaling cascade may be involved in fluke outgrowth. Further investigation into LMX1B and EN‐1 in the flukes is needed to confirm this working hypothesis about the WNT7A results.

The epithelial and mesenchymal SHH signaling within the flukes shows variation craniocaudally and between the two tissue types (Figure 7A,C). Like the other proteins, SHH signals were present in the epithelium of all sections. However, SHH staining was unusual in that punctate speckling was found throughout the ligamentous layer of mesenchyme in all sections of fluke tissue. This SHH staining in the epithelium and mesenchyme appeared much darker in the distal portion of the fluke compared to the lighter staining near the peduncle, indicating the presence of greater amounts of protein towards the distal end of the flukes. SHH staining within the mesenchyme is also found in two distinct hotspots within the ligamentous and core layer along the distal aspect of the fluke (Figure 7C, 1 and 2) but tearing in the mesenchyme on the left side interrupts this pattern, making interpretation difficult. These two hotspots are only found on this section, and staining of slides just proximal and distal to this section did not reveal the same hotspots. SHH is found throughout the developing embryos of all mammals and plays a critical role in establishing differential cell fates within multiple organ systems. The immunohistochemical (IHC) data presented here, particularly the presence of two hotspots, suggest that SHH is likely serving a morphogenetic function in the beluga flukes as well. Further testing of SHH regulators (GLI proteins) and receptors (PTCH1) in the hedgehog signaling pathway will provide greater insight into SHH activity in the cetacean flukes.

In a typical limb, GREM is found between the ZPA and AER. In the beluga flukes, the GREM antibody stained within the ligamentous layer of the mesenchyme and within the epithelium, similar to the SHH staining pattern (Figures 8 and 9). The GREM data for the flukes do not resemble the expected pattern for appendage signaling. Unlike the more discrete regionalization found in developing limbs of other tetrapods including mice, cichlid fish, catfish, and zebrafish,^43^, ^53^, ^98^, ^99^, ^100^, ^101^ the GREM staining in the flukes was diffuse throughout the mesenchyme. Within the beluga flukes, it is possible that GREM is promoting and maintaining the feedback loop between FGF8 and SHH, as has been demonstrated in limb research,^98^, ^99^, ^102^ but direct testing of this hypothesis is needed.

Dolphins have evolved a mechanism for maintaining their interdigital webbing via co‐expressed FGF, BMP, and GREM signals in the interdigital tissue.^73^ We tested the beluga flukes for BMP4 and found no evidence of protein expression within any of the tissues (data not shown) but do see evidence of overlapping GREM and FGF signaling within the epithelial tissue of the flukes.

In summary, the protein data from the flukes offer broad support for the appendage hypothesis. In the strictest sense, only FGF8 was found in the expected location for an AER; the other four proteins were reported in different regions or tissue layers than expected for complete homology with fin or limb expression. However, the presence of all five proteins within the flukes provides some evidence that generalized relationships between these proteins are preserved within the flukes. These data highlight the importance of homology and recapitulation in the context of the evolution of a novel appendage.

### 3.3 Novelty and homology of the flukes

While our data set precludes any information on causality of the proteins studied, the locality and relationships of the FGFs, SHH, and GREM are all reminiscent of other appendage studies. In this respect, our data are similar to many other protein studies conducted in appendages and highlights the deep homology of the limb patterning paradigm. The flukes resemble a highly modified soft‐tissue appendage, akin to cetacean flippers.

Flukes do not form until completion of somitogenesis within the tail. Caudal somites come from unique paraxial mesoderm which is initially formed from a neuromesodermal bipotential stem cell in the tail bud.^103^, ^104^, ^105^, ^106^ While there is overlap in the expression patterns of body somites and tail somites, tail somites are a molecularly distinct tissue when compared to the trunk.^107^, ^108^, ^109^, ^110^ Given our evidence that the flukes utilize a signaling pattern similar to limbs, this suggests that all of the proteins found in the mesenchyme are being expressed by paraxial, not lateral plate, mesoderm in the cetacean tail. This translocation of gene expression to a different germ layer and tissue type is similar to median fin development in the catshark (*Scyliorhinus canicula*), where it has been experimentally demonstrated that the dorsal fin arises from paraxial mesoderm and utilizes the same gene cascades as the lateral plate derived pectoral fins.^111^ Additionally, the olive flounder (*Paralichthys olivaceus*) demonstrated molecular markers for cells derived from neural crest and paraxial mesoderm in the dorsal fin.^112^ The flounder dorsal fin expressed mRNA for *SHH*, *WNT7A*, and *FGF8* in overlapping regions, similar to what is being observed in the flukes.

Overall, our data suggest that cetacean flukes are a morphologically novel organ reusing a highly conserved signaling pathway. The presence of FGF8, FGF10, WNT7A, SHH, and GREM within the flukes all point to an appendage‐like signaling cascade deriving from the tetrapod limb bud. This work provides a key insight into the evolution and development of a novel organ.

### 3.4 Experimental procedure

Three beluga embryos, NSB‐DWM 2009LDL9F, 2014LDL7F, and 2012LDL10F were used for analysis. These beluga embryos were collected as part of the Iñupiat beluga harvest that occurs in Point Lay, Alaska, for both cultural and subsistence fulfillment.^113^, ^114^ All specimens were collected under NOAA‐NMFS permit 17,350. These embryos correspond to Carnegie Stage 19, 20, and the fetal period as described in Gavazzi et al.^27^ All three specimens were paraffin‐sectioned for histology cranially to caudally. This sectioned fluke tissue was subjected to multiple histological stains and immunohistochemistry.

### 3.5 Hematoxylin and eosin, Mallory's trichrome

Two stains were used to identify cell types and internal morphology of the embryonic fluke tissue. Modified Harris hematoxylin in acetic acid and 1% eosin Y diluted in 95% ethanol were first used to identify basic tissue types. A modified Mallory's trichrome, taken from Everett and Miller^84^ provided additional information. This trichrome is adapted for embryonic tissue, which can be difficult to stain due to the undifferentiated state of many organs. The protocol uses 1% aqueous acid‐fuscin red, 0.5% aniline blue in 8% acetic acid, and 2% orange G in 8% acetic acid to distinguish between tissue types. Red blood cells stain red, nuclei stain orange, undifferentiated connective tissue is blue while differentiated connective tissue is blue‐purple. Muscle, epithelial tissue, and cytoplasm will stain purple, and the protocol also states that highly proliferative and metabolically active cells will take on an orange hue throughout the entire cell, not just the nuclei.

### 3.6 Immunohistochemistry

To assess the importance of common embryonic proteins in fluke outgrowth, we performed a modified immunohistochemical (IHC) protocol that was used in other cetacean protein signaling research^73^, ^88^, ^115^, ^116^ on one beluga embryo, NSB‐DWM 2009LDL9F. Primary antibodies were incubated overnight at 4°C, the secondary antibody was incubated for 2 h at room temperature, and the avidin‐biotin reagent was incubated for 90 min at room temperature. The tissue was exposed to 3,3′‐diaminobenzidine (DAB) for 8 min and then counterstained with 0.01% thionine for contrast. Concentrations of the antibodies were as follows: FGF8 at 1:100 (Invitrogen: PA5‐79295), FGF10 at 1:750 (Invitrogen: PA5‐88291), SHH at 1:100 (Invitrogen: PA5‐19492), WNT7A at 1:200 (Invitrogen: PA5‐80231) and GREM 1:100 (Invitrogen: PA5‐121945). The antibody for BMP4 (Invitrogen: PA5‐27288) was tested at the following concentrations: 1:50, 1:100, 1:200, 1:250, 1:500, 1:750, and 1:1000. No antigen binding and subsequent chromogen staining was detected at any of these concentrations for BMP4.

## FUNDING INFORMATION

This work was supported by the Kent State University Graduate Student Senate Research Award and Hennecke Family Foundation.
